# Engineered *in-vitro* cell line mixtures and robust evaluation of computational methods for clonal decomposition and longitudinal dynamics in cancer

**DOI:** 10.1038/s41598-017-13338-8

**Published:** 2017-10-18

**Authors:** Hossein Farahani, Camila P. E. de Souza, Raewyn Billings, Damian Yap, Karey Shumansky, Adrian Wan, Daniel Lai, Anne-Marie Mes-Masson, Samuel Aparicio, Sohrab P. Shah

**Affiliations:** 1 BC Cancer Agency, Department of Molecular Oncology, Vancouver, V5Z 1L3 Canada; 2 University of British Columbia, Department of Pathology and Laboratory Medicine, Vancouver, V6T 2B5 Canada; 3 BC Cancer Agency, Michael Smith Genome Sciences Centre, Vancouver, V5Z 1L3 Canada; 4 Centre de recherche du Centre hospitalier de l’ Université de Montréal (CRCHUM), Montreal, Canada; 5 Institut du cancer de Montréal, Montreal, Canada; 6 Department of Medicine, Université de Montréal, Montreal, Canada

## Abstract

Characterization and quantification of tumour clonal populations over time via longitudinal sampling are essential components in understanding and predicting the response to therapeutic interventions. Computational methods for inferring tumour clonal composition from deep-targeted sequencing data are ubiquitous, however due to the lack of a ground truth biological data, evaluating their performance is difficult. In this work, we generate a benchmark data set that simulates tumour longitudinal growth and heterogeneity by *in vitro* mixing of cancer cell lines with known proportions. We apply four different algorithms to our ground truth data set and assess their performance in inferring clonal composition using different metrics. We also analyse the performance of these algorithms on breast tumour xenograft samples. We conclude that methods that can simultaneously analyse multiple samples while accounting for copy number alterations as a factor in allelic measurements exhibit the most accurate predictions. These results will inform future functional genomics oriented studies of model systems where time series measurements in the context of therapeutic interventions are becoming increasingly common. These studies will need computational models which accurately reflect the multi-factorial nature of allele measurement in cancer including, as we show here, segmental aneuploidies.

## Introduction

Computational decomposition of human cancers into constituent clonal populations is a major goal of investigators seeking to measure and interpret clonal dynamics in tumours. Enumerating, characterizing and quantifying distinct cancer clonal populations within a tumour constitute essential steps toward elucidating properties governing disease natural histories and response to therapeutic intervention. The temporal growth of these cancer clonal populations measured via longitudinal sampling can be performed to a limited degree in patients through primary-relapse comparisons^1,2^ and in much finer granularity in pre-clinical studies through serial engraftment of patient material in immunocompromised mice^3^ or in passaged cell lines. Furthermore, recent developments in circulating tumour DNA (ctDNA) technology will decrease the need for invasive biopsies and consequently result in increased availability of longitudinal time series data from patients. Through longitudinal sampling, one can in theory measure the dynamic abundance of genomically defined clones through digital counting capacities of next generation sequencing devices, providing a framework to study relative fitness properties of differentiated clones. However, one can at best only represent variant allele fraction by direct interpretation of the resultant read data. To account for confounding factors of sample preparation, non-malignant cell populations and copy number alterations (CNA), many computational methods have been developed for mapping variant allele fraction (VAF) to clonal prevalence (or variously mutation cellular prevalence or cancer cell fraction). These methods^4–14^ vary in their approaches (outlined in Table 1) according to type of input data, assumptions about phylogenetic processes and incorporation of copy number alterations in their inference model. The efficacy of these methods for identifying mutation clusters as markers of clones have not been rigourously evaluated in the context of longitudinal sampling, nor have any comparative studies been performed where ground truth about clonal population architecture and dynamics is known.

**Table 1:** Algorithms to infer clonal/cluster composition and their properties/assumptions.

Algorithm/Property	Input data	Model/approach	CNA	Phylogenetic inference	Multiple samples
**Clomial** ^4^	DTS^1^	non-Bayesian generative Binomial	N^2^	N	Y^3^
**SciClone** ^5^	DTS	Bayesian Beta mixture	Y	N	Y
**PyClone** ^6^	DTS	Dirichlet process, Beta-Binomial	Y	N	Y
**PhyloWGS** ^7^	WGS^4^ and DTS	Tree-stick-breaking process, Binomial	Y	Y	Y
TrAp^8^	CP^5^	deterministic search under constraints	I^6^	Y	N
LICHeE^9^	VAF^7^, CP	perfect phylogeny model	I	Y	Y
Rec-BTP^10^	VAF, CP	binary tree partition	I	Y	N
CITUP^11^	VAF, CP	combinatorial algorithm	I	Y	Y
SubCloneSeeker^12^	CP	exaustive tree enumeration	I	Y	Y
PhyloSub^13^	DTS	predecessor of PhyloWGS without phylogenic correction for CNA	Y	Y	Y
CloneHD^14^	WGS	HMM^8^, variational bayes	Y	N	Y

^1^Deep-targeted sequencing; ^2^no; ^3^yes; ^4^whole genome sequencing; ^5^celular prevalence; ^6^indirectly via CP; ^7^variant allele frequency; ^8^hidden Markov model.

In this work we provide a benchmark data set that simulates tumour longitudinal growth of cancer clones *in vitro* using physical and controlled mixtures of cancer cell lines at known proportions. Our data consist of deep targeted sequenced reference and variant read counts from a set of single nucleotide variant (SNV) positions from mixtures of both diploid and aneuploid cancer cell lines with known genomic landscape. Because the true clonal architecture of real tumour samples is always unknown, our cell mixing data provide a more realistic model than synthetic data to assess the performance of different computational methods. In this work we present a comparison of the performance of PyClone^6^, Clomial^4^, SciClone^5^, and PhyloWGS^7^ (algorithms in bold in Table 1) on our ground truth datasets. We selected these four algorithms because they can be applied directly and simultaneously to all of our deep-targeted data samples. In addition to the mixture of cell lines we also analyze the performance of PyClone, Clomial, SciClone and PhyloWGS on breast tumour xenograft samples from the case SA494 studied by^3^ as for this case the authors provide single-cell data validating their results. To further study the strengths and limitations of these algorithms we conduct subsampling studies where we apply each algorithm to our cell mixing data downsampling read depth, number of SNV positions and number of samples.

## Results

### Selection and validation of data set

**Figure 1:**
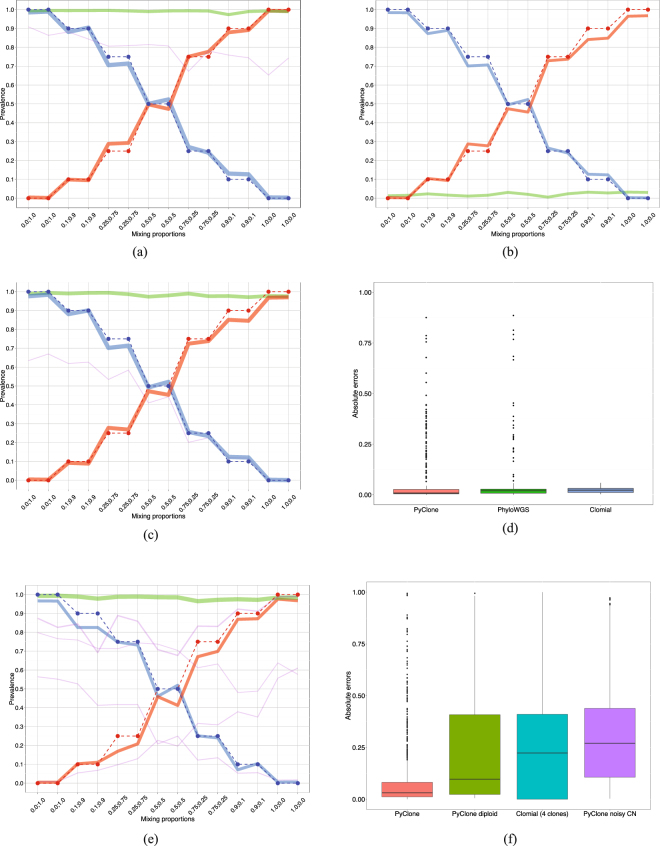
Results for Experiment 1 and 2. (**a**) *Experiment 1* (*diploid cell lines HCT116 and 184*-*hTERT*-*L2*). PyClone predicted cluster prevalences for each mixture. The vertical axis indicates the range of cluster prevalence. The horizontal axis indicates the true mixing proportions used to generate each sample, where x;y corresponds to proportion of 184-hTERT-L2 and HCT116, respectively. The dashed red and blue lines represent the true simulated tumour longitudinal growth for 184-hTERT-L2 and HCT116, respectively, and the solid lines show the predicted longitudinal growth given by the estimated cluster prevalences. The red solid line corresponds to a cluster containing 41 184-hTERT-L2 specific SNVs, the blue solid line to a cluster of 48 HCT116 specific SNVs, the green solid line to a cluster of 33 shared SNVs and the purple solid line to small cluster containing three shared SNVs. (**b**) *Experiment 1* (*diploid cell lines HCT116 and 184*-*hTERT*-*L2*). Clomial predicted clonal prevalences for each mixture. The red, blue and green solid lines correspond to the predicted clonal prevalences for 184-hTERT-L2, HCT116 and normal clones, respectively. The other plot components are as in (**a**). (**c**) *Experiment 1* (*diploid cell lines HCT116 and 184*-*hTERT*-*L2*). PhyloWGS predicted cluster prevalences for each mixture. The red, blue and green solid lines correspond to the predicted cluster prevalences for 184-hTERT-L2, HCT116 and shared cluster, respectively. The other plot components are as in (**a**). (**d**) *Experiment 1* (*diploid cell lines HCT116 and 184*-*hTERT*-*L2*). Box plots of the absolute difference between estimated and true SNV cellular prevalences across all samples for PyClone, PhyloWGS and Clomial. (**e**) *Experiment 2* (*aneuploid cell lines DAH55 and DAH56*). PyClone predicted cluster prevalences. The horizontal and vertical axes are as in (**a**). The dashed red and blue lines represent the true simulated tumour longitudinal growth for DAH55 and DAH56, respectively. The solid lines show the PyClone predicted cluster prevalences. The red solid line corresponds to a cluster containing 25 DAH55 specific SNVs, the blue solid line to a cluster of 24 DAH56 specific SNVs, the green solid line to a cluster of mainly shared SNVs and the purple solid lines to four other small clusters. (**f**) *Experiment 2* (*aneuploid cell lines DAH55 and DAH56*). Box plots of the absolute difference between estimated and true SNV cellular prevalences across all samples for PyClone using the correct copy number information, PyClone assuming diploid loci, Clomial and PyClone with noisy copy numbers.

To represent longitudinal tumour growth we designed two experiments. In each experiment, DNA extracted from two different cancer cell lines were mixed at various proportions forming a total of 14 samples (see Table 2). By ordering these mixture proportions as in Table 2, we are simulating the longitudinal growth of a tumour where one clonal population (e.g., red dotted line in Fig. 1(a)) expands while the other one (e.g., blue dotted lines in Fig. 1(a)) shrinks. The mixed samples were subjected to deep-targeted sequencing on 144 target SNV positions (see Fig. S1(a) of the Supplementary Information and Experiment details). Primers were designed to specifically amplify targeted regions of the genome which surround these unique SNVs that identify the individual cell lines in the cell mixing model that we propose. In order for an accurate representation of the allelic prevalence, we sequenced those targeted regions deeply achieving a median coverage of 5488 and 11754 reads for Experiments 1 and 2, respectively. The cell lines used in this project each had orthogonally derived bulk exome and copy number data. In Experiment 1 we used the HCT116 and 184-hTERT-L2 cell lines as they are regarded as being nearly diploid (see^15^ and^16^, respectively). The cell line HCT116 was derived from the colon of an adult male with colorectal carcinoma and the 184-hTERT-L2 cell line was derived from human mammary epithelial cells immortalized by transduction with hTERT. For Experiment 2 we chose the ovarian cancer cell lines TOV3133D and TOV3133G (see^17^). These are cell lines derived from one individual, are copy number complex (see Fig. S1(b,c) of the Supplementary Information) and thus provide a more biologically relevant model for solid epithelial cancers with genomic instability. The cell lines TOV3133D and TOV3133G will be referred to as DAH55 and DAH56, respectively.

**Table 2:** Mixing proportions of cell lines in Experiment 1 and Experiment 2.

Mixture ID*	Cell line A	Cell line B
2	0	1
14	0	1
7	0.1	0.9
19	0.1	0.9
6	0.25	0.75
18	0.25	0.75
5	0.5	0.5
17	0.5	0.5
4	0.75	0.25
16	0.75	0.25
3	0.9	0.1
15	0.9	0.1
1	1	0
13	1	0

In Experiment 1, A and B correspond to the 184-hTERT-L2 and HCT116 cell lines, respectively. In Experiment 2, A and B correspond to DAH55 and DAH56, respectively.

*As per laboratory protocol.

An initial list of potential target heterozygous SNV positions were selected for each cell line by aligning exome sequences and calling SNVs. For each pair of cell lines A and B three sets of 48 target heterozygous SNVs were selected: one specific to cell line A, one specific to cell line B and one with SNVs shared between both cell lines. We then statistically validated each target position (see Section 2 of the Supplementary Information) obtaining a final list of targets for each experiment. For Experiment 1 we obtained 48 SNVs specific to the HCT116 cell line, 42 SNVs specific to 184-hTERT-L2 and 38 shared ones. For Experiment 2 our validation resulted in 33 DAH55 specific SNVs, 33 DAH56 specific and 39 shared ones. See Table S1(a) and (b) of the Supplementary Information for their genomic coordinates under the hg19 genome build. In addition, Table S1(a) and (b) contain the variant and reference counts for each SNV per sample for Experiments 1 and 2, respectively. Figure S2(a–d) of the Supplementary Information show the VAFs for the SNVs considering 100% mixtures (i.e., pure samples) for both experiments. Histograms of the VAFs for one of the mixed samples (mixture 6 in Table 2) for Experiments 1 and 2 can be found in Fig. S2(e,f) of the Supplementary Information, respectively. The expectation is that each set of SNVs should be clustered together with shared prevalences close to the true mixing mixing proportions in the output of any analysis from the algorithms described below.

### Overview of algorithms

We focus on single nucleotide variants (SNVs) and, therefore, define a sub-population clone by a set of cells sharing the same mutational profile. Some algorithms infer the number and prevalence of clones in a tumour sample(s), while other algorithms try to cluster mutations either by their cellular prevalences or by their variant allele frequencies.

PyClone^6^, is hierarchical Bayesian statistical model for estimating cellular prevalences from deeply targeted sequenced somatic mutations followed by clustering them based on the estimated cellular prevalences. PyClone considers allelic imbalances generated by copy number alterations and normal-cell contamination. It estimates the parameters of interest via MCMC methods. The algorithm outputs a posterior density for each mutation’s cellular prevalence and a similarity matrix containing the probability of any two mutations occurring in the same cluster. Two mutations are assigned to the same cluster if they occur at very similar cellular prevalence in the sample(s).

Zare and co-authors^4^ propose Clomial, a generative Binomial model that incorporates the allelic frequencies of a set of somatic mutations from multiple tumour samples to infer the prevalences and genotypes of a specified number of clones. The authors assume that all mutations are at heterozygous and diploid loci. The parameters of interest are estimated via the Expectation-Maximization (EM) algorithm^18^ assuming independence of the samples, independence of the mutations and non-zero normal cell contamination at each sample. In order to choose the number of clones, the authors propose using a method such as the Bayesian Information Criterion (BIC)^19^.

Miller and co-authors^5^ introduce SciClone, a method for estimating the number and composition of clusters of mutations across one or more samples. SciClone uses an approach based on a variational Bayesian Beta mixture model to cluster primarily variant allele frequencies (VAFs) from heterozygous and diploid loci. The method automatically infers the number of clusters, however, in contrast to PyClone it does not estimate the cellular prevalence of each mutation. Although the focus of the algorithm is copy number neutral loci, Miller *et al*. claimed that integration of copy number altered loci is possible. However, our results show that mutations in aneuploid loci are automatically not assigned to any of the clusters and sometimes even SNVs in diploid regions cannot be assigned to any cluster.

Deshwar and colleagues^7^ propose PhyloWGS, a non-parametric Bayesian method to cluster SNVs and infer tumour phylogenetic trees. Unlike his predecessor PhyloSub^13^, PhyloWGS introduces a phylogenic correction for VAFs in loci in regions with copy number alterations. PhyloWGS employs an MCMC method for inference. This method can be applied to a single sample or to multiple samples simultaneously, however, in case of multiple samples, the input copy number information for each SNV position has to be the same across all samples or alternatively aneuploid in one sample and diploid in remaining ones. Because in our Experiment 2 each SNV position has copy number information varying across samples we can only apply PhyloWGS to each single sample separately. For Experiment 1, where all targets in all samples are in diploid regions, we can apply PhyloWGS simultaneously to all samples.

### Experiment 1: mixture of diploid cell lines

In Experiment 1 there are three ground truth clusters of targeted positions: one for HCT116 cell line, another one for 184-hTERT-L2 cell line, and a third cluster composed of the shared targets between these two cell lines. In addition the prevalences corresponding to the HCT116 and 184-hTERT-L2 clusters follow the simulated longitudinal tumour growth, i.e., the true mixing proportions across samples.

We applied PyClone to all 14 mixture samples in Experiment 1 simultaneously with all final 128 target SNV positions having copy number two and tumour content of 100%. Figure 1a shows the inferred PyClone clusters and their estimated prevalences (solid lines) along with the true simulated longitudinal tumour growth (red and blue dashed lines corresponding to 184-hTERT-L2 and HCT116, respectively). The prevalence of a cluster in a given sample is obtained by calculating the median of the posterior cellular prevalence means of the mutations in that cluster. PyClone inferred three major clusters (blue, red and green solid lines in Fig. 1a) with estimated prevalences close to the expected ones. All SNV positions specific to cell line HCT116 are correctly assigned to the same cluster. Except by one target, all SNVs specific to 184-hTERT-L2 are in the same cluster. Another major cluster was obtained containing only shared mutations. A few smaller clusters were also formed: one with three shared SNV positions and three mono-clusters (see Table S2 and the co-clustering plot in Fig. S3(a) of the Supplementary Information).

We applied Clomial to Experiment 1 data considering three, four and five clones and used the BIC score proposed and implemented in^4^ to choose the best model. Based on this criterion, we chose three clones (see Table S3 of the Supplementary Information): a clone of normal cells (normal clone), a cell line HCT116 clone and a cell line 184-hTERT-L2 clone. In this scenario the clonal prevalences learned by Clomial can be plotted in the same way as PyClone cluster prevalences, however, the green solid line now corresponds to the normal clone. Because our samples consist of only cancer cells, we expect the normal clone prevalences to be very close to zero. Figure 1b shows that the inferred clonal prevalences by Clomial for each cell line are close to true mixing proportions. All SNVs were assigned to their correct clone (see Fig. S3(b) of the Supplementary Information).

SciClone was applied to all samples in this experiment and it performed reasonably. Table S4 of the Supplementary Information shows that the target SNVs are assigned into four clusters, however SciClone could not assign 25 SNVs to any cluster. Cluster 1 consists of only shared positions. Clusters 2 and 3 correspond to cell lines HCT116 and hTERT-184, respectively. Figure S3(c) of the Supplementary Information depicts its co-clustering plot for Experiment 1.

We also applied PhyloWGS simultaneously to all 14 samples in Experiment 1 considering all targets across all samples in diploid copy number regions. PhyloWGS inferred three major clusters (blue, red and green solid lines in Fig. 1c) with estimated prevalences close to the true ones. All shared targets are correctly assigned to one major cluster. Except by one target, all mutations specific to 184-hTERT-L2 are in the same cluster. Another major cluster contains only HCT116 specific targets. One small cluster containing two HCT116 specific target positions was also obtained (purple solid line in Fig. 1c) along with two mono-clusters (see Table S5 and Fig. S3(d) of the Supplementary Information).

In order to compare the accuracy of PyClone, Clomial and PhyloWGS in estimating the mutation cellular prevalences, we calculated the absolute prevalence errors, that is, the absolute difference between the estimated and true cellular prevalence values for each SNV in Experiment 1. Figure 1d shows the absolute errors across all samples for each algorithm. The median absolute error and interquartile ranges are presented in Table S6 of the Supplementary Information. It is important to recall that SciClone does not infer mutation prevalences and, therefore, cannot be included in this comparison. We can observe in Fig. 1d the presence of outliers in the box plots corresponding to PyClone and PhyloWGS, these outliers correspond to SNVs that were assigned to wrong clusters leading to estimated prevalences far from the true ones. Because Clomial assigns all SNVs to the correct clusters we do not observe any outliers in the distribution of its absolute errors. We can also conclude that PyClone leads to absolute errors that are significantly smaller than the ones corresponding to Clomial considering a nonparametric statistical test robust to outliers (pairwise one-sided Wilcoxon rank sum test with correction for multiple testing, *p*-value < 2 × 10^−16^).

We considered the V-measure^20^ to compare the clustering performance of PyClone, Clomial, PhyloWGS and SciClone. The V-measure represents the homogeneity and completeness of a clustering procedure result. To satisfy the homogeneity criterion, a clustering procedure must assign only those SNVs that are members of a single group to a single cluster. Completeness is symmetrical to homogeneity and in order to satisfy the completeness criterion, a clustering method must assign all of those SNVs that are members of a single group to a single cluster. The weighted harmonic mean of homogeneity and completeness gives rise to the V-measure. In the optimal case where a clustering procedure assigns all SNVs to their correct groups the V-measure is one.

Table 3 shows that in Experiment 1 the clustering performance of Clomial is optimal and the best among all algorithms with V-measure equals to one.

**Table 3:** V-measure clustering performance of PyClone, Clomial, SciClone and PhyloWGS. In parentheses homogeneity and completeness, respectively.

Algorithm	Experiment 1	Experiment 2 (correct copy numbers)
PyClone	0.92 (1.0; 0.85)	0.63 (0.78; 0.52)
Clomial	1.0 (1.0; 1.0)	—
SciClone	0.72 (0.84; 0.65)	—
PhyloWGS	0.94 (1.0; 0.88)	—

### Experiment 2: mixture of aneuploid cell lines

Experiment 2 contains the data corresponding to the aneuploid cancer cell lines DAH55 and DAH56. Below we present the results of applying PyClone to these data as this is the only algorithm that can be applied to our multi-sample data simultaneously while allowing each SNV to have a different copy number in each sample.

In this experiment we applied PyClone to all 14 samples with tumour content of 100% and sample specific SNV copy numbers calculated by averaging the copy numbers obtained from SNP6 data (see Methods) for each cell line according to the sample mixing proportions. Similar to Experiment 1, PyClone also inferred three major clusters with estimated prevalences close to expected ones (see Fig. 1e). One major cluster is composed of 25 DAH55 specific SNVs, another one of 24 DAH56 specific SNVs, and a third one of mainly shared SNVs. Besides the three major clusters there were four other small clusters and one mono-cluster (see Table S7 and Fig. S4(a) of the Supplementary Information). The orange box plot in Fig. 1f shows the absolute errors in estimating the mutation cellular prevalences across all samples for PyClone considering the correct copy number information. Regarding the clustering performance PyClone shows a V-measure equals to 0.63 (Table 3).

**Figure 2:**
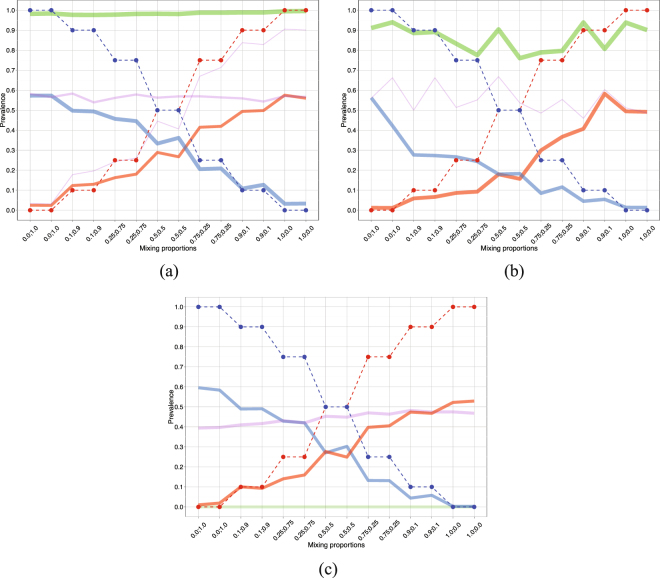
Experiment 2 (aneuploid cell lines DAH55 and DAH56). Effect of using incorrect copy numbers. (**a**) PyClone predicted cluster prevalences assuming diploid loci. The solid lines show the PyClone predicted cluster prevalences. The red solid line corresponds to a cluster of 21 DAH55 specific SNVs, the blue solid line to 27 DAH56 specific SNVs, the green solid line to a cluster of mainly shared SNVs and the purple solid lines to two other clusters, one of size 17 and the other of size 8 (see Supplementary Table S13). The other plot components are as in Fig. 1e. (**b**) PyClone predicted cluster prevalences adding random noise to copy number information. The solid lines show the PyClone predicted cluster prevalences. The red solid line corresponds to a cluster of 29 DAH55 specific SNVs, the blue solid line to 27 DAH56 specific SNVs, the green solid line to a cluster of 34 mainly shared SNVs and the purple solid line to a cluster of 9 mainly shared SNVs (see Supplementary Table S14). The other plot components are as in Fig. 1e. (**c**) Clomial predicted clonal prevalences. The red, blue, and purple solid lines correspond to the predicted clonal prevalences for the three estimated clones and the green solid line corresponds to the normal clone (see Tables S15 and S16 of the Supplementary Information).

To study the importance of using the correct copy number information in PyClone, we maintained the same variant and reference read counts while assuming all SNVs are in diploid loci. We also applied PyClone to Experiment 2 data perturbing the copy number information by randomly adding or subtracting copies from each SNV. Figure 2a,b show that using incorrect copy numbers greatly deteriorates the performance of PyClone as estimated prevalences are far from the true mixing proportions. Figure S4(b,c) of the Supplementary Information present the corresponding co-clustering plots.

Although Clomial is built for samples with diploid targeted positions, we also applied it to the data from Experiment 2 in order to investigate the effects of assuming inaccurate copy number information in clonal inference. We considered three, four and five clones and based on the BIC score (see Table S8 of the Supplementary Information) four clones were chosen instead of the correct answer of three clones (see Fig. 2c). We also applied SciClone to the data from Experiment 2 assuming diploid loci obtaining eight different clusters (see Table S9 of the Supplementary Information).

Figure 1f shows the box plots of the absolute errors in estimating the mutation cellular prevalences across all samples for PyClone using the correct copy number information, PyClone assuming diploid loci, PyClone with noisy copy numbers as well as Clomial with four inferred clones. Table S10 of the Supplementary Information presents the median value of the absolute errors and the interquartile ranges for each of these approaches. We observe that PyClone with correct copy numbers leads to absolute errors that are significantly smaller than the ones corresponding to PyClone assuming diploid loci, PyClone with noisy copy numbers or Clomial (pairwise one-sided Wilcoxon rank sum test with correction for multiple testing, *p*-values < 2 × 10^−16^). Table S11 of the Supplementary Information contains the corresponding V-measure, homogeneity and completeness scores for each approach considered in Experiment 2.

### Performance of the algorithms on breast tumour xenograft samples

In addition to the mixtures of cell lines we also assess the performance of PyClone, Clomial, SciClone on breast tumour xenograft samples from case SA494 studied by Eirew *et al*. in^3^. In contrast to our cell mixture samples, the true clonal composition of the bulk tumour and xenograft samples is unknown. In order to validate their findings from deep-targeted sequencing on bulk DNA, Eirew *et al*. also performed targeted sequencing at single cell resolution. We consider their single cell data as ground truth and used them to measure the performance of each algorithm.

**Figure 3:**
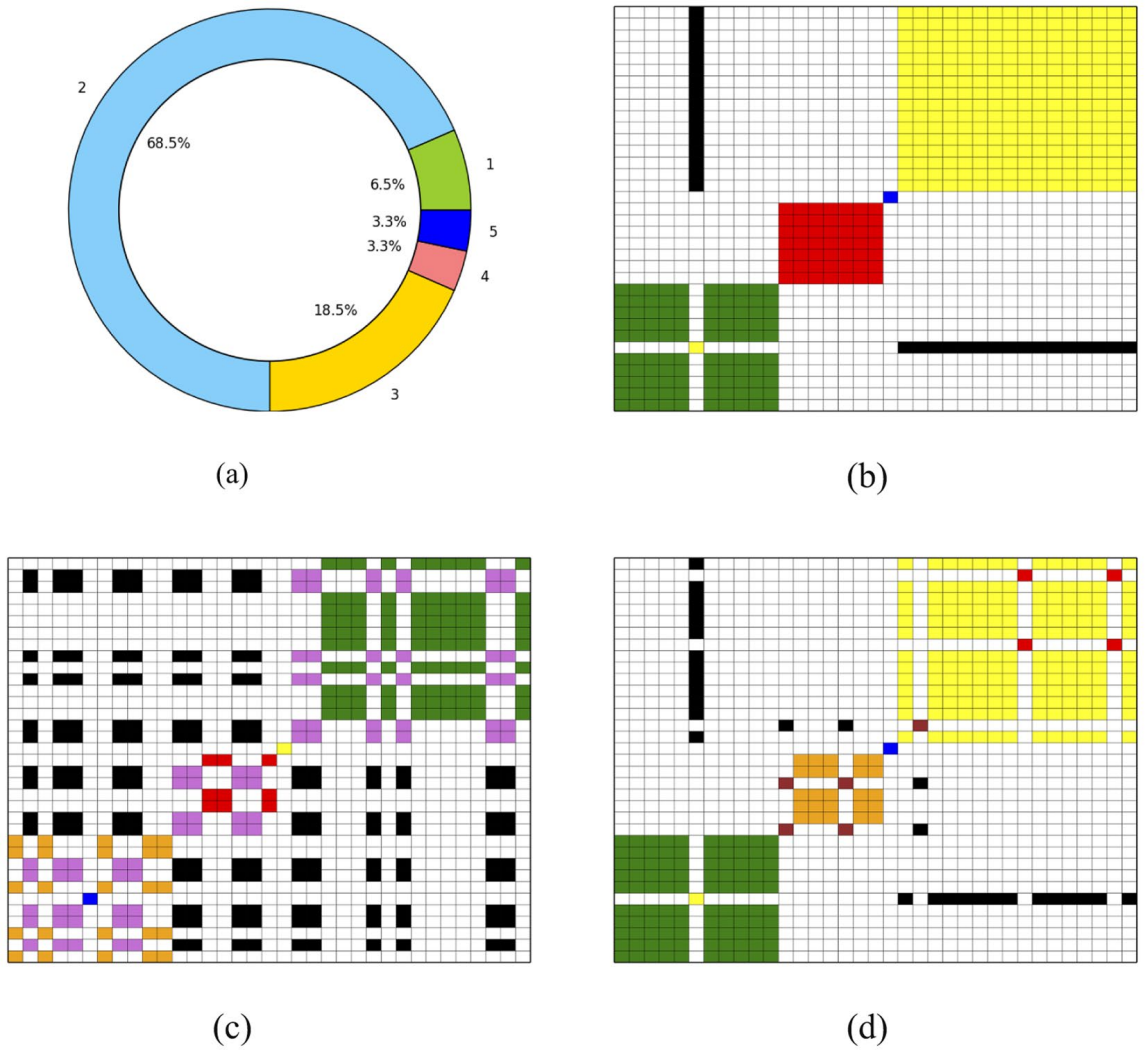
Performance of the algorithms on a real world data set validated with deep targeted sequencing data at single nuclei resolution from^3^. **(a)** Distribution of copy numbers across the targeted SNVs. (**b**–**d**) Co-clustering performance of PyClone, SciClone and Clomial, respectively.

The bulk deep-sequenced data for case SA494 comprises of reference and variant allele counts for 89 SNV target positions along with their major and minor copy number information (see Fig. 3a) from one tumour sample and three xenograft passages. We applied PyClone, Clomial and SciClone to all 89 SNV targets in the SA494 data set simultaneously for all samples. A subset of the original 89 SNVs were used by Eirew *et al*. in their targeted-deep sequencing experiment at single cell resolution in 42 isolated tumour nuclei and 56 isolated nuclei from a xenograft passage. Using Bayesian phylogenetic inference Eirew *et al*. found that two major clones of nuclei emerged in the SA494 case, one comprising tumour nuclei and the other xenograft nuclei. By considering the presence and absence of the SNVs in each nuclei based on a threshold for VAFs we can conclude that there are 17 targets that are shared between tumour and the fourth passage xenograft. There are 11 targets specific to the tumour and 7 targets specific to the fourth passage xenograft. Using these results as the ground truth, we assessed the performance of each algorithm via co-clustering plots.

Figure 3b–d show the co-clustering performance of PyClone, SciClone and Clomial, respectively. The performance of these algorithms on tumour and xenograft data follows the same pattern as their performance on the cell mixture data. Figure 3b shows that PyClone only misclassifies two out of the 35 SNVs verified by single nuclei sequencing. Figure 3d presents the results of applying Clomial to the data considering three clones. We can observe in Fig. 3d that six SNVs were misclassified. SciClone delivers the worst performance being unable to assign 15 SNVs to any cluster and assigning 15 SNVs to the wrong cluster (see Fig. 3c).

### Subsampling studies

In order to further study the strengths and limitations of these algorithms we conducted subsampling studies where we apply PyClone, Clomial, PhyloWGS and SciClone to our cell mixing samples simultaneously downsampling read depth, number of targeted SNVs and number of samples (see Methods).

**Figure 4:**
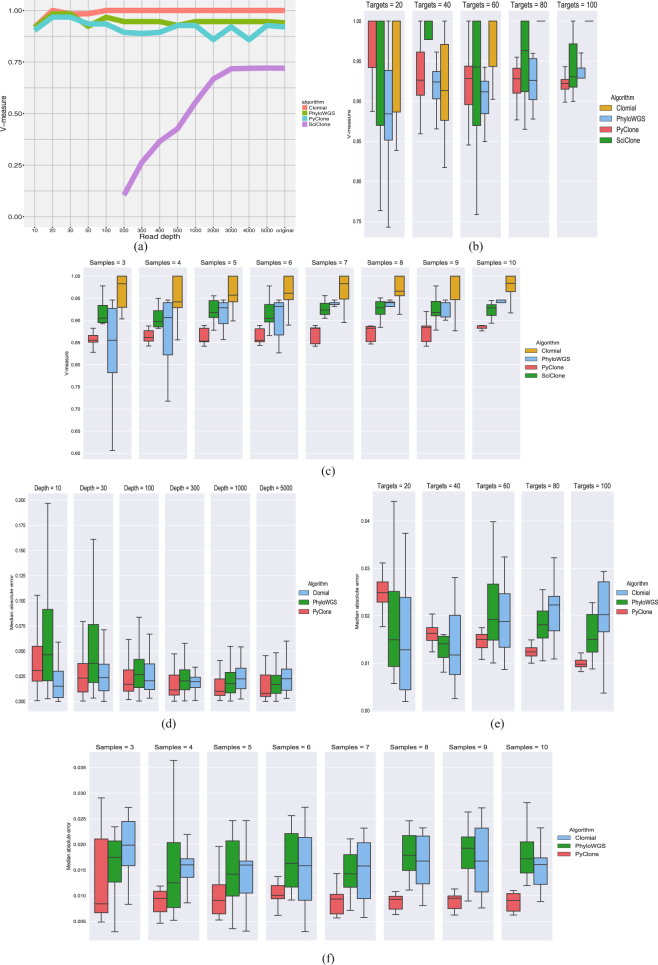
Results of simulation studies for Experiment 1 (diploid cell lines HCT116 and 184-hTERT-L2). (**a**) *Experiment 1*, *V*-*measure*, *downsampling read depth*. The vertical axis indicates the V-measure score obtained by each algorithm for each read depth considered when downsampling the number of variant and reference reads aligned to each SNV. (**b**) *Experiment 1*, *V*-*measure*, *downsampling the number of targets*. Box plots of the V-measure scores obtained by applying each algorithm to each simulated data set under each number of targeted SNVs considered. Note that outliers were omitted from the box plots. (**c**) *Experiment 1*, *V*-*measure*, *downsampling the number of samples*. Box plots of the V-measure scores obtained by applying each algorithm to each simulated data set for each possible number of samples considered. Note that outliers were omitted from the box plots. (**d**) *Experiment 1*, *prevalence error*, *downsampling read depth*. Box plots of the absolute errors in estimating cellular prevalence obtained by applying each algorithm to each read depth considered. Note that outliers were omitted from the box plots. (**e**) *Experiment 1*, *prevalence error*, *downsampling number of targets*. Box plots of the median absolute errors obtained by applying each algorithm to each simulated data set under each number of targeted SNVs considered. Note that outliers were omitted from the box plots. (**f**) *Experiment 1*, *prevalence error*, *downsampling number of samples*. Box plots of the median absolute errors obtained by applying each algorithm to each simulated data set under each number of samples considered. Note that outliers were omitted from the box plots.

Figure 4a–c show how the V-measure changes in Experiment 1 when downsampling the number of reads, number of targeted SNVs and number of samples, respectively. We can observe in Fig. 4a that SciClone is the only algorithm showing very small V-measure values when decreasing read depth and it actually does not converge when read depth is equal or smaller than one hundred. The other algorithms do not show great changes in V-measure when varying read depth. Regarding downsampling the number of targets, Fig. 4b shows that increasing the number of targets leads to smaller values of V-measure for PyClone whereas for Clomial it leads to larger V-measure values. Because PyClone is a non-parametric Bayesian clustering method increasing the number of SNVs tends to increase the number of unnecessary cluster splits, which harms completeness and, therefore, decreases the V-measure score. Figure 4c shows that the clustering performance of PhyloWGS is greatly improved by increasing the number of samples, however, the same pattern cannot be observed for the other algorithms.

We also assessed how the error in estimating mutational cellular prevalences changes when downsampling the read depth, number of SNVs and number of samples (see Fig. 4d–f, respectively). We observe in Fig. 4d large errors in estimating cellular prevalence for read depth values smaller than one hundred. However, we do not observe a great change in error values when read depth is greater or equal than one hundred. Regarding downsampling the number of targets, Fig. 4e shows that PyClone leads to smaller errors as the number of targets increase. The same pattern is not observed for the other algorithms. We can observe in Fig. 4f that increasing the number of samples leads to smaller error variability for PyClone.

**Figure 5:**
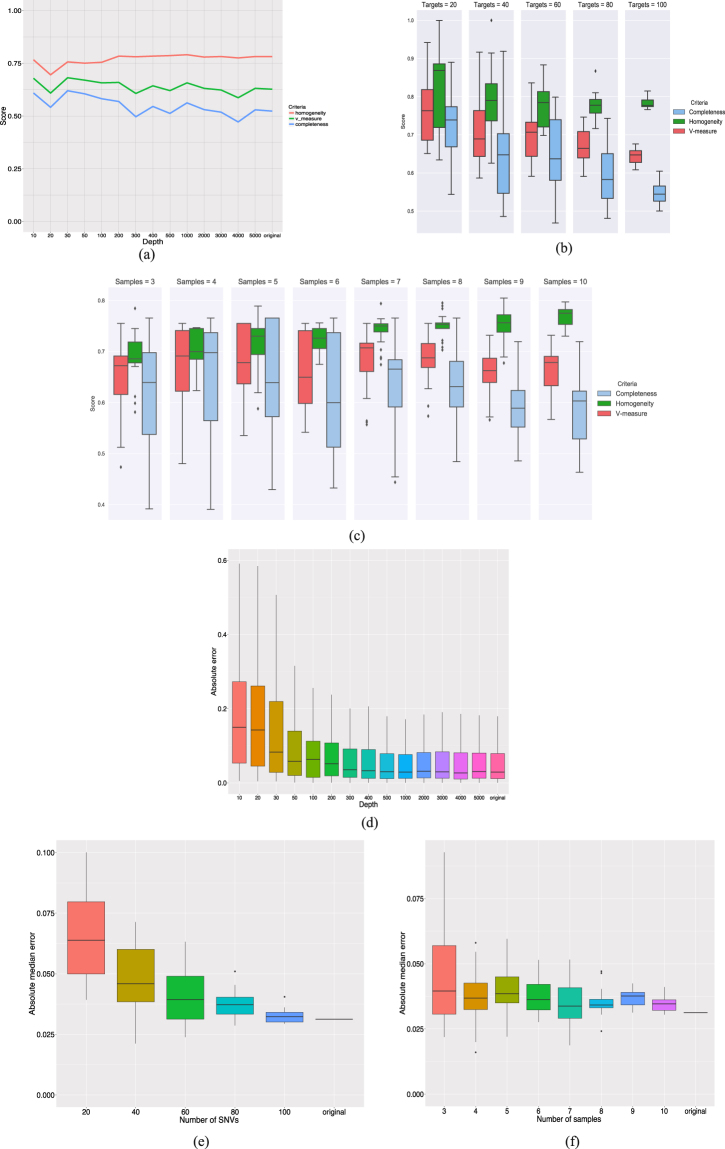
Results of simulation studies for Experiment 2 (aneuploid cell lines DAH55 and DAH56). (**a**) *Experiment 2*, *V*-*measure*, *downsampling read depth*. The vertical axis indicates the V-measure, homogeneity and completeness scores obtained by applying PyClone for each read depth considered when downsampling the number of variant and reference reads aligned to each SNV. (**b**) *Experiment 2*, *V*-*measure*, *downsampling the number of targets*. Box plots of the V-measure, homogeneity and completeness scores obtained by applying PyClone to each simulated data set under each number of targeted SNVs considered. (**c**) *Experiment 2*, *V*-*measure*, *downsampling the number of samples*. Box plots of the V-measure, homogeneity and completeness scores obtained by applying PyClone to each possible number of samples considered. (**d**) *Experiment 2*, *prevalence error*, *downsampling read depth*. Box plots of the absolute errors in estimating cellular prevalence obtained by applying PyClone to each read depth considered. Note that outliers were omitted from the box plots to facilitate visualization of the median, first and third absolute error quartiles. (**e**) *Experiment 2*, *prevalence error*, *downsampling number of targets*. Box plots of the median absolute errors obtained by applying PyClone to each simulated data set under each number of targeted SNVs considered. (**f**) *Experiment 2*, *prevalence error*, *downsampling number of samples*. Box plots of the median absolute errors obtained by applying PyClone to each simulated data set under each number of samples considered.

In the simulation studies using Experiment 2 data we considered only PyClone as this is the only algorithm that can be applied simultaneously to all samples with each SNV having different copy number information across samples. Figure 5a shows that read depth does not have a great impact in PyClone clustering performance measured via V-measure. Regarding the number of targets, as in the results for Experiment 1, we observe smaller values of completeness and, therefore, smaller values of V-measure when the number of SNVs gets larger (see Fig. 5b). We can observe in Fig. 5c that increasing the number of samples does not have a great impact in the V-measure scores, however, it does improve homogeneity.

Figure 5d–f show the impact in cellular prevalence estimation caused by downsampling read depth, number of targets and number of samples, respectively. As in Experiment 1, Fig. 5d shows large errors when read depth is smaller than one hundred. We observe in Fig. 5e that increasing the number of targets leads to smaller errors. We can also observe smaller variability in the errors by increasing the number of samples (see Fig. 5f).

In addition to the results described above, in Section 3 of the Suplementary Information we present the results of applying PyClone, SciClone and PhyloWGS to the data of each single sample in our experiments separately. Clomial was not considered in this single sample analysis as it can only be used when the number of samples is at least equal to the number of clones due to constraint problems.

## Discussion

Measuring and modeling dynamics in cancer cell populations from longitudinal sampling is paramount to understanding the properties and patterns leading to clinical endpoints such as treatment resistance. As the field moves toward a “population genetics” framework for ascribing quantitative fitness attributes to genotypes under interventional selection, a necessary first step is to accurately measure the abundance of genetically distinct populations. Here, we show that correct computational inference of the prevalence of clones in experimentally simulated time series next generation sequencing data must account for copy number alterations present in aneuploid cells. We generated a dataset from controlled admixtures of two cell line pairs to mimic growth trajectories of two clones where one clone grows from low abundance to saturation at the expense of the other. As the cells are real experimental reagents, this dataset represents the closest real’ground truth’ dataset in the field and provides a substrate for further computational tool development and benchmarking. Importantly, it will complement *in silico* datasets which often fall short of capturing all sources of experimental variation and thus may only sub-optimally represent the properties of datasets observed in practice. Using this dataset, we show through quantitative comparisons of methods representing mutation clustering and phylogenetics inspired models that inference of both cancer cell fraction and correct clustering of mutations are highly dependent on the consideration of copy number state of the allele being measured. This is best exemplified through comparison of both diploid and aneuploid cell mixtures. In diploid scenarios, prevalence estimates are quite accurate for the models we tested. However in the aneuploid setting, performance was significantly better for the model which incorporated correct prior copy number information. These results will inform future functional genomics oriented studies of model systems where time series measurements in the context of therapeutic interventions are becoming increasingly common. As we showed in patient derived xenografts, temporal sampling can reveal important properties of clonal evolution including reproducible clonal dynamics^3^. The advent of genetic intervention screens using CRISPR/Cas9 and other related systems will undoubtedly then benefit from measuring clonal dynamics to interpret fitness and selection characteristics. Finally, we expect our results will inform time series modelling from patients using non-invasive techniques such as measuring alleles in cell-free ctDNA in plasma. Advances in ctDNA technology opens the opportunity for non-invasive longitudinal monitoring of tumour burden in patients as a function of treatment regimens. All of these (and other) experimental designs will need to leverage computational models which accurately reflect the multi-factorial nature of allele measurement in cancer including, as we show here, segmental aneuploidies. In summary, we provide a dataset substrate and a framework for ground-truth based evaluation of models for the field as it continues its progress towards routine measurement of cancers as dynamic, and evolving systems.

## Methods

### Experiment details

#### Cell culture

The 184-hTERT cell lines were cultured at 37 °C, 5% CO_2_, in serum-free mammary epithelial cell basal media (MEBM, Lonza), supplemented with mammary epithelial cell growth media single quots (Lonza), 5 *μ*g/ml transferrin (Sigma), 1.25 M of isoproterenol (Sigma Aldrich). HCT116 cell lines were cultured at 37 °C, 5% CO_2_, in McCoys 5A media (Sigma Aldrich) supplemented with 10% FBS (Sigma Aldrich). The TOV3133D and TOV3133G cell lines were cultured at 37 °C, 5% CO_2_, in a 1:1 mix of media 199 (Sigma Aldrich) and media 105 (Sigma Aldrich) supplemented with 10% FBS.

#### Cell mixing

Cell mixing was carried out according to a sample’s DNA concentration. DNA was extracted from pelleted cells and quantified. The extracted cell proportions were then mixed together according to DNA concentration. For example in order to calculate how much sample is required for each mix all samples are to be at 5 ng/*μ*l per reaction for 150 reactions, (5 × 150 = 750 ng in total). In order to calculate mixing amounts: first the calculation of concentration was required for each mix. 0.9 (90% of 750 ng) = 675 ng and 0.1 (10% of 750 ng) = 75 ng. Then the required amount of sample for the mix was calculated, If 0.9 sample had a DNA concentration of 482 ng/*μ*l, then 675/482 = 1.40 *μ*l of sample was added, and if 0.1 sample had a DNA concentration of 8.8 ng/*μ*l then 75/8.8 = 8.5 *μ*l sample was added to mix. The required sample volumes were mixed and re-suspended with TE buffer to obtain the required volume for qPCR.

#### Exome data alignment and SNV calling

Exome sequences were aligned using BWA and SNVs were called using Samtools in Experiment 1 and MutationSeq^21^ in Experiment 2.

#### PCR primer design and primer selection

The 2-Step PCR sequencing method used primers that were designed as singleplex primers. Chosen target positions were entered into Primer3, an online program used for primer design. (Primer3 File - http://primer3.sourceforge.net). See Section 2 of the Supplementary Information for Primer3 specific settings. After Primer3 design all selected target primers were validated with in-silico PCR using the UCSC online program (http://genome.ucsc.edu/cgi-bin/hgPcr). Targets positions that passed all design QC were used. In order for primer compatibility with the MiSeq chemistry, adapter sequences were added to each primer. Sequence information was supplied by Illumina. Primers with the Illumina adapter sequence were ordered desalted from IDT at 0.5 nM concentration, final volume 250 *μ*l.

All primers that were singleplex-designed were tested for amplification performance using the qPCR method described below. PCR products were also run on a 2% agarose gel (Sigma Aldrich) to check the size of the band and quality of each primer pair. If primers passed all QC checks, they were used in the experiments. In total 48 primers for cell line 1, 48 primers for cell line 2 and 48 primers shared across cell lines 1 and 2 were selected. Table S12a and b in the Supplementary Information contain selected primers and corresponding sequences for Experiments 1 and 2, respectively.

#### Molecular biology techniques

DNA was extracted using the QIAamp DNA mini kit (Qiagen), using the protocol for cultured cells. DNA was eluted with 20 *μ*l elution buffer to increase DNA concentration. DNA concentration and quantitation was measured by flourometry using Qubit, dsDNA BR Assay (Life Technologies) per the manufacturer’s protocol. DNA quality was assessed using the NanoDrop ND1000 (ThermoScientific) with 1 ul of extracted genomic DNA as per the manufacturers protocol.

qPCR was performed with 5 ng of genomic DNA template, 5 *μ*l SYBR Select Mastermix 2x (Life Technologies), and 0.2 *μ*M each of forward and reverse primers. Each primer pair was performed as a singleplex reaction. Cycling conditions were as follows: Standard curve (AQ), 50 °C for 2 min, followed by 40 cycles of [95 °C for 10 s, 95 °C for 15 s, 60 °C for 1 min]. A dissociation step was also added to the end of the program. The ABI 7900HT was used for all qPCR experiments.

The PCR for the 2-step MiSeq method was performed with 1 *μ*l of the qPCR ExoSAP DNA template, 10x FastStart HiFi Rxn buffer w/o MgCl_2_, 25 mM MgCl_2_, DMSO, 10 ml PCR grade Nucleotide, 5 U/*μ*l FastStart HiFi Enzyme (all from Roche), and 4 *μ*l of each I7 and I5 Barcode-Adapters (Illumina). PCR cycling conditions were as follows: 95 °C for 10 min, followed by 15 cycles of [95 °C for 15 s, 60 °C for 30 s, 72 °C for 1 min, 72 °C for 3 min] and 4 °C hold.

The Bioanalyser was used as a quality control step for determining the correct size distribution of the SPRIselect and magnetic bead purified samples, and then pooled as one sample for MiSeq sequencing. Quality control and size distribution of the samples was performed using the Agilent DNA 1000 DNA kit (Agilent Technologies) per the manufacturer’s protocol yielding to 1 *μ*l of the sample required.

#### Amplicon library construction - Singleplex PCR sequencing method

After samples were mixed, 5 ng total of genomic DNA was used per reaction for each sample. The protocol used for amplicon library construction and Singleplex PCR sequencing is the same as in^3^.

#### Deep targeted data alignment and SNV calling

Deep targeted sequences were aligned using BWA and SNVs were called using MutationSeq^21^ in both experiments.

#### Experiment 2 copy number information

Copy number information for Experiment 2 was obtained from OncoSNP-SEQ^22^ analysis of the DAH55 and DAH56 cell line copy number array measurements (SNP6).

### Subsampling studies

In order to downsample the total number of reads of each SNV in a particular sample to a certain read depth, say 10x, we proceeded as follows. First we computed the average total number of reads across all SNVs in that sample. Next we calculated what proportion of the average total number of reads the desirable read depth of 10x corresponds to. For each SNV we multiply this proportion to its total read counts obtaining the downsampled read depth, and we then randomly and uniformly sample this downsampled number of reads from all variant and reference reads corresponding to that SNV. As a result we obtain an average read depth across all SNVs of 10x. We then applied each algorithm to each downsampled data set.

To downsample the number of SNVs in our data we considered various total number of target SNV positions (20, 40, 60, 80 and 100 targets) and proceeded as follows. For each possible number of targets we generated 20 simulated data sets by randomly picking the desirable number of targets. For example, for 40 targets, we generated 20 simulated data sets by randomly picking 40 targets from our original target list for each data set. We then applied the different algorithms to each data set under each possible number of targets considered.

In order to downsample the number of samples considering different number of samples (from 3 to 10) we did as follows. For each possible number of samples we randomly generated 20 sets of data with that number of samples. So, for example, for three samples we randomly generated 20 combinations of 3 samples from our total of 14 samples making sure all combinations were different. We then applied the different algorithms to each data set under each possible number of samples considered.

### Software information

PyClone 0.13.0 available from http://compbio.bccrc.ca/software/pyclone.

Clomial 1.3.0 available at R-bioconductor.

SciClone available from https://github.com/genome/sciclone.

PhyloWGS available from https://github.com/morrislab/phylowgs.

## Electronic supplementary material

Supplementary Information

Supplementary Table S1a

Supplementary Table S1b

Supplementary Table S2

Supplementary Table S3

Supplementary Table S4

Supplementary Table S5

Supplementary Table S6

Supplementary Table S7

Supplementary Table S8

Supplementary Table S9

Supplementary Table S10

Supplementary Table S11

Supplementary Table S12a

Supplementary Table S12b

Supplementary Table S13

Supplementary Table S14

Supplementary Table S15

Supplementary Table S16
