# Supplementary material for: Engineered in-vitro cell line mixtures and robust evaluation of computational methods for clonal decomposition and longitudinal dynamics in cancer
Source: Sci Rep. 2017 Oct 18;7:13467. doi: 10.1038/s41598-017-13338-8 (PMC5647443; doi:10.1038/s41598-017-13338-8)
Supplement: Supplementary file 1 — Supplementary Information [file 41598_2017_13338_MOESM1_ESM.pdf]

Engineered in-vitro cell line mixtures and robust evaluation of computation methods for clonal decomposition and longitudinal dynamics in cancer

Hossein Faharani, Camila P. E. de Souza, Raewyn Billings, Damian Yap, Karey Shumansky, Adrian Wan, Daniel Lai, Anne-Marie Mes-Masson, Samuel Aparicio and Sohrab Shah

Supplementary Information

1 Description of the supplementary tables

The following supplementary tables are available for this manuscript.

Table S1a (S1a.xls): Genomic coordinates of targeted SNVs for Experiment 1 along with cell line group membership and number of reference and variant read counts per each sample.

Table S1b (S1b.xls): Genomic coordinates and copy numbers of targeted SNVs for Experiment 2 along with cell line group membership and number of reference and variant read counts per each sample.

Table S2 (S2.xls): PyClone estimated clusters along with estimated prevalences for Experiment 1.

Table S3 (S3.xls): Clomial BIC scores for Experiment 1.

Table S4 (S4.xlsx): Distributions of SNVs in the clusters learned by SciClone in Experiment 1.

Table S5 (S5.xls): PhyloWGS estimated clusters along with estimated prevalences for Experiment 1.

Table S6 (S6.xlsx): Median absolute errors and interquartile ranges (IQR) in estimating mutation prevalences for each algorithm in each experiment.

Table S7 (S7.xls): PyClone estimated clusters along with estimated prevalences for Experiment 2.

Table S8 (S8.xls): Clomial BIC scores for Experiment 2 assuming diploid loci.

Table S9 (S9.xlsx): Distributions of SNVs in the clusters learned by SciClone in Experiment 2 assuming diploid loci.

Table S10 (S10.xlsx): Median absolute errors and interquartile ranges (IQR) in estimating mutation prevalences for each algorithm in Experiment 2.

Table S11 (S11.xlsx): V-measure, homogeneity and completeness scores for each algorithm in Experiment 2.

Tables S12a and S12b (S12a.xls and S12b.xls): Selected primers and corresponding sequences for Experiments 1 and 2, respectively.

Table S13 (S13.xls): PyClone estimated clusters along with estimated prevalences for Experiment 2 assuming diploid loci.

Table S14 (S14.xls): PyClone estimated clusters along with estimated prevalences for Experiment 2 with noisy copy numbers.

Table S15 (S15.xls): Clomial clonal prevalences for Experiment 2 assuming diploid loci - four clones were chosen.

Table S16 (S16.xls): Clomial estimated clonal composition for Experiment 2 assuming diploid loci - four clones were chosen.

2 Supplementary information

2.1 Statistical procedure for validation of SNV target positions

In order to validate and obtain the final list of SNV target positions for our experiments we proceeded as follows. For each amplicon region we used the number of reads with reference and variant alleles for each position to calculate a background variant probability, we then used a binomial test with this probability to obtain a p-value for the significance of the target SNV in that region. To obtain the final list of SNVs specific to only one of the cell lines we applied our binomial test to all amplicon regions in the two replicate mixtures with 100% that cell line (see Table 2 of the main text). We selected the positions with p-value $< 10^{-16}$ in both 100% mixtures that intersect with the initial list of positions for that cell line. In order to obtain the final list of shared SNV positions, first we take again the intersection of the positions with p-value $< 10^{-16}$ in the two 100% replicates for each cell line obtaining two lists, we then found the intersection between these two lists and the original list of chosen shared target positions.

2.2 Primer3 specific settings

```
P3_FILE_TYPE=settings
PRIMER_THERMODYNAMIC_PARAMETERS_PATH=/meta/o/oncoapop/Apps/primer3-2.3.5/src/primer3_config/
PRIMER_MISPRIMING_LIBRARY=/meta/o/oncoapop/Apps/primer3-
2.3.5/src/humrep_and_simple.txt PRIMER_LIBERAL_BASE=0
PRIMER_LIB_AMBIGUITY_CODES_CONSENSUS=0 PRIMER_MIN_SIZE=18
PRIMER_OPT_SIZE=22 PRIMER_MAX_SIZE=25 PRIMER_MIN_TM=57.0
PRIMER_OPT_TM=59.0
PRIMER_MAX_TM=63.0 PRIMER_PAIR_MAX_DIFF_TM=5.0
PRIMER_PRODUCT_SIZE_RANGE=150-170 140-190 PRIMER_EXPLAIN_FLAG=1
PRIMER_NUM_RETURN=5 PRIMER_MAX_NS_ACCEPTED=0
PRIMER_MAX_POLY_X=4 PRIMER_GC_CLAMP=2
PRIMER_MIN_LEFT_THREE_PRIME_DISTANCE=5
PRIMER_MIN_RIGHT_THREE_PRIME_DISTANCE=5
```

2.3 Selected primers

See Tables S12a and S12b.

3 Subsampling results for single samples

Figures 5 and 6 show the V-measure scores obtained when downsampling the number of targets and read depth, respectively, and applying SciClone and PyClone to each single sample in Experiment 1 separately. We observe in 5 that PyClone presents greater V-measure scores than SciClone. We also observe in 6 that decreasing read depth leads to smaller V-measure values and that SciClone could not provide any results when read depth was smaller than one hundred.

Figures 7 and 8 present the V-measure scores obtained when downsampling the number of targets and read depth, respectively, and applying PyClone to each single sample in Experiment 2 separately. We can observe that smaller V-measure scores when the number of targets increase and higher median V-measure scores when read depth increases.

Figures 9 and 10 show that the absolute prevalence errors produced by PyClone decrease as the number of targets and read depth increases.

Figure 11 shows the absolute errors in estimating mutation prevalence when applying PhyloWGS to the original data of each sample in Experiment 1 and 2 separately. We observe that the diploid samples in Experiment 1 lead to much smaller errors than the aneuploid samples in Experiment 2.

Figure legends

Figure 1: *Experimental pipeline and Experiment 2 copy number data.* **(a)** Experimental pipeline. In both experiments, DNA is extracted from both cell lines and mixed according to the desired proportion. After the library construction the mixtures are sequencing using MiSeq.
(b) and **(c)** show the proportion of targeted SNVs at loci with k total copy number for DAH55 (TOV3133D) and DAH56 (TOV3133G) cell lines, respectively.

Figure 2: *VAF plots.*
(a) Scatter plot of the VAFs from samples 1 and sample 2 in Experiment 1. Because sample 1 corresponds to a mixture with 100% 184-hTERT-L2 cell lines we observe that all VAFs for the hTERT specific SNVs (green dots) are spread across the x-axis and because sample 2 corresponds to the 100% HCT116 cell line mixture we see all VAFs for the HCT116 specific SNVs (red dots) spread across the y-axis. The VAFs corresponding to the shared SNVs (blue dots) between both cells lines appear in the middle of the plot.
(b) Scatter plot of the VAFs from samples 13 and sample 14 in Experiment 1.
(c) Scatter plot of the VAFs from samples 1 and sample 2 in Experiment 2.
(d) Scatter plot of the VAFs from samples 13 and sample 14 in Experiment 2.
(e) Histogram of the VAFs from sample 6 in Experiment 1 (25% 184-hTERT-L2 and 75% HCT116).
(f) Histogram of the VAFs from sample 6 in Experiment 2 (25% DAH55 and 75% DAH56).

Figure 3: *Experiment 1 (diploid cell lines HCT116 and 184-hTERT-L2).*
(a) Co-clustering performance of PyClone. This figure shows the performance of PyClone in correctly assigning each SNV to its corresponding true cluster (HCT116, 184-hTERT-L2 and shared). Each inferred cluster corresponds to a different color.
(b) Co-clustering performance of Clomial.
(c) Co-clustering performance of SciClone. SNVs that are not correctly assigned to their correct cluster are in black.
(d) Co-clustering performance of PhyloWGS.

Figure 4: *Experiment 2 (aneuploid cell lines DAH55 and DAH56).*

(a) Co-clustering performance of PyClone using the correct copy number information.

(b) Co-clustering performance of PyClone assuming diploid loci.

(c) Co-clustering performance of PyClone with noisy copy numbers.

Figure 5: *V-measure scores when downsampling the number of targets in Experiment 1 and applying PyClone (red box plots) and SciClone (green box plots) to each sample separately.*

Figure 6: *V-measure scores when downsampling the number of reads in Experiment 1 and applying PyClone and SciClone to each sample separately.*

Figure 7: *V-measure scores when downsampling the number of targets in Experiment 2 and applying PyClone to each sample separately.*

Figure 8: *V-measure scores when downsampling the number of reads in Experiment 2 and applying PyClone to each sample separately.*

Figure 9: *PyClone median absolute errors in cellular prevalence estimation when downsampling the number of targets for single samples in Experiment 1.*

Figure 10: *PyClone median absolute errors in cellular prevalence estimation when downsampling read depth for single samples in Experiment 2.*

Figure 11: *Median absolute errors in cellular prevalence estimation when applying PhyloWGS to the original data of each sample in Experiments 1 (red box plots) and 2 (green box plots) separately.*

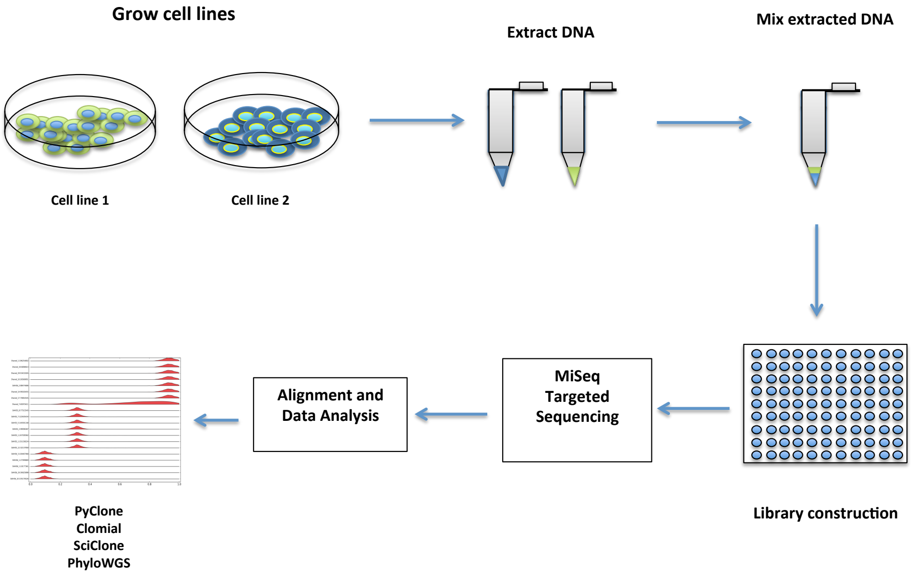
Grow cell lines
Cell line 1
Cell line 2
Extract DNA
Mix extracted DNA
Library construction
MiSeq Targeted Sequencing
Alignment and Data Analysis
PyClone
Clomial
SciClone
PhyloWGS

(a)

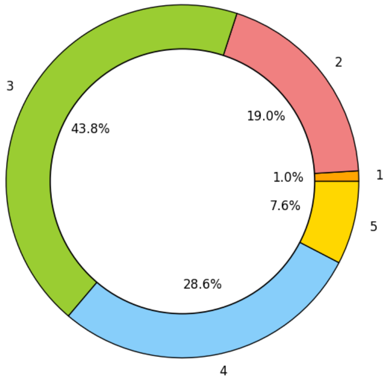
3
43.8%
2
19.0%
1
1.0%
7.6%
5
28.6%
4

(b)

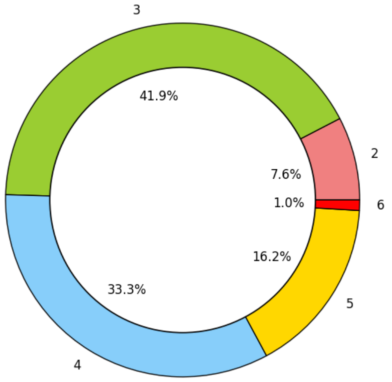
3
41.9%
2
7.6%
1.0%
6
16.2%
5
33.3%
4

(c)

Figure 1

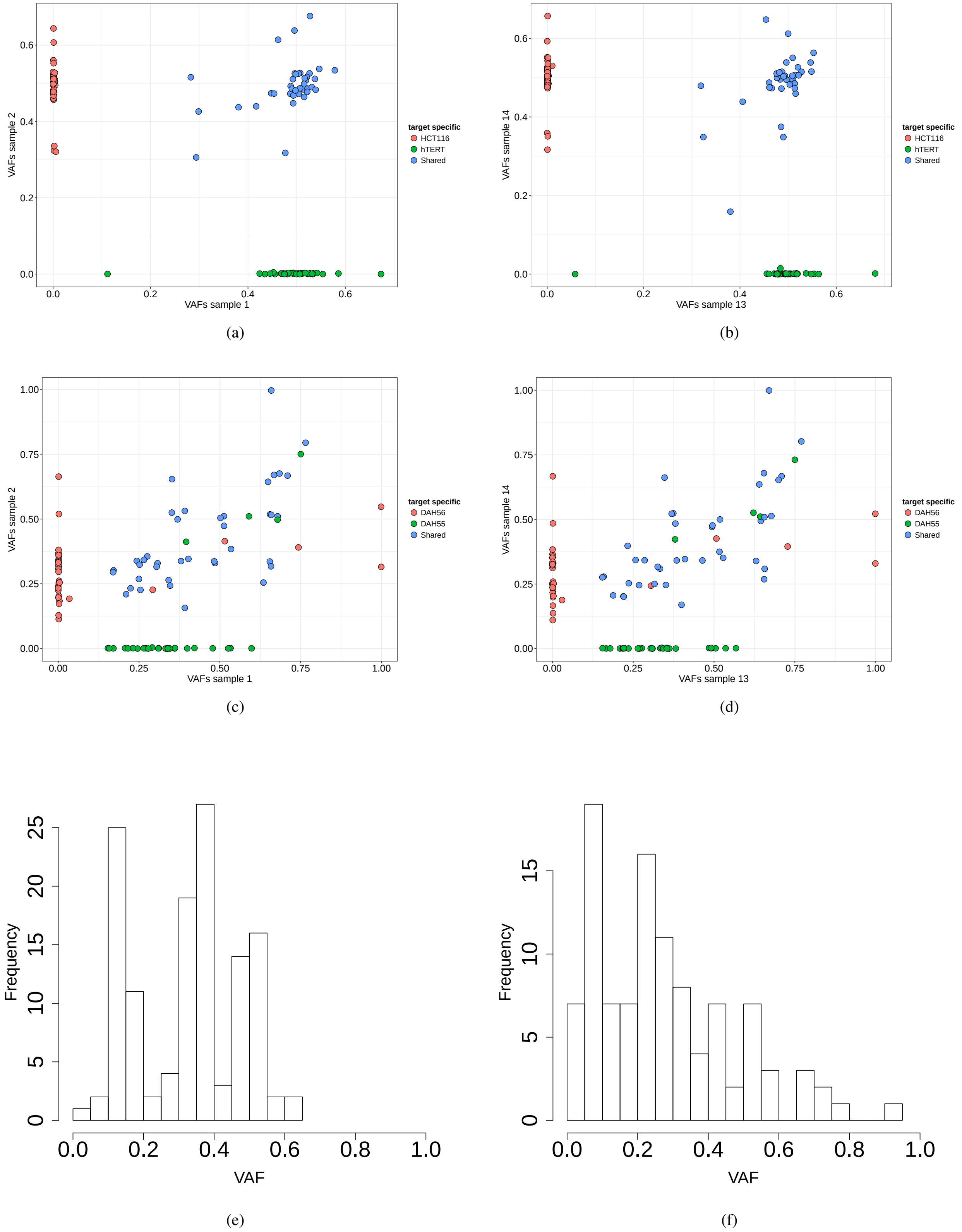

(a)
VAFs sample 2
VAFs sample 1
target specific
HCT116
hTERT
Shared
(b)
VAFs sample 14
VAFs sample 13
target specific
HCT116
hTERT
Shared
(c)
VAFs sample 2
VAFs sample 1
target specific
DAH56
DAH55
Shared
(d)
VAFs sample 14
VAFs sample 13
target specific
DAH56
DAH55
Shared
(e)
Frequency
VAF
(f)
Frequency
VAF

Figure 2

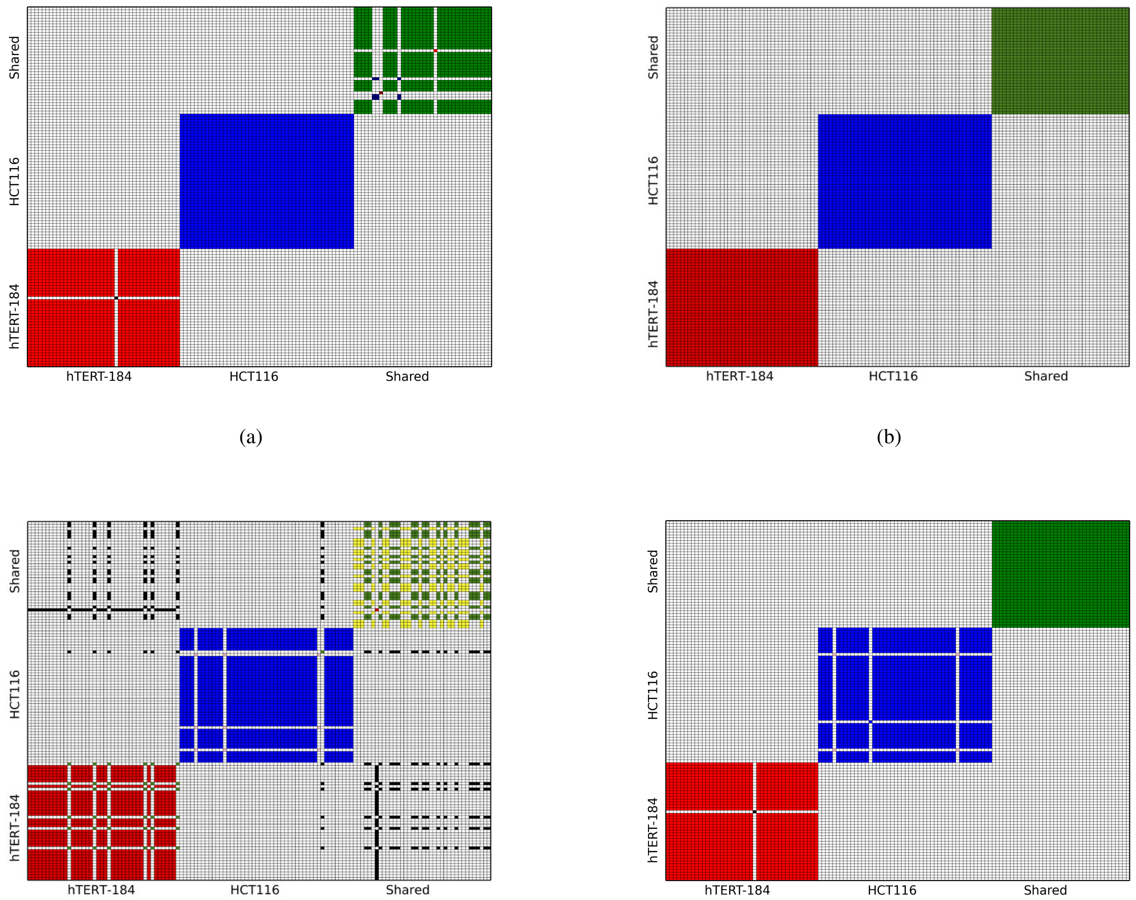

(a)

(b)

(c)

(d)

Figure 3

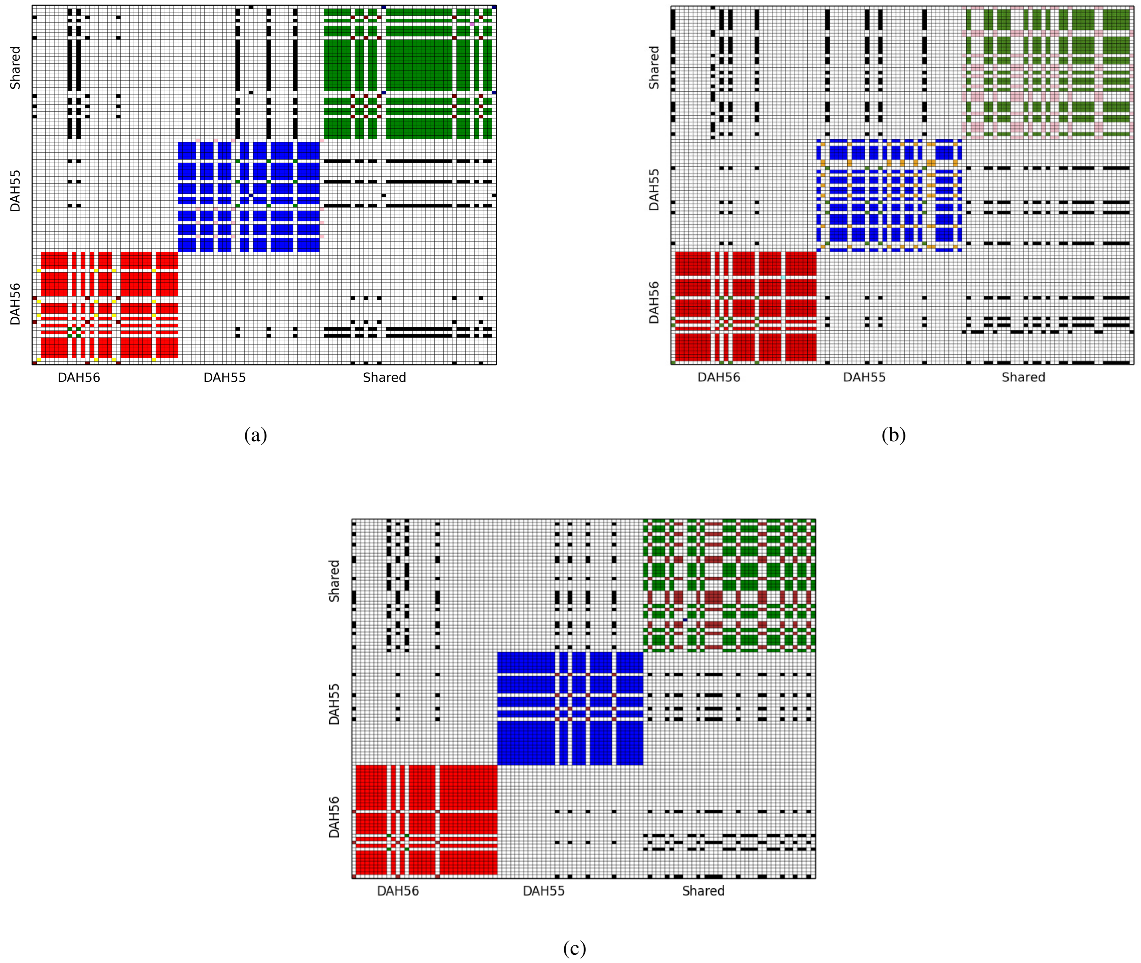

Figure 4

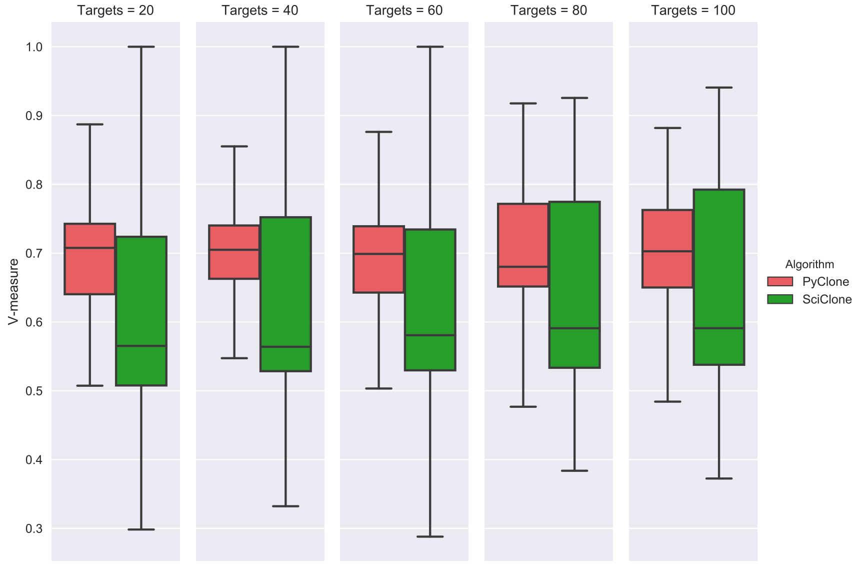

Figure 5

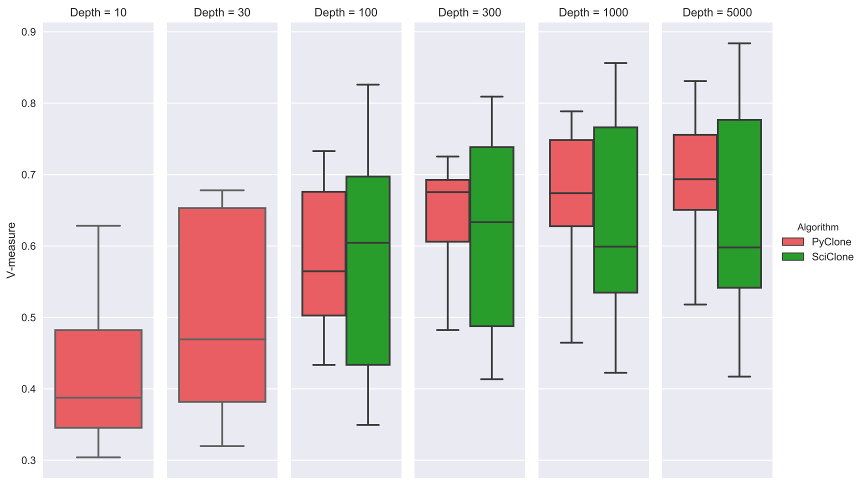

Figure 6

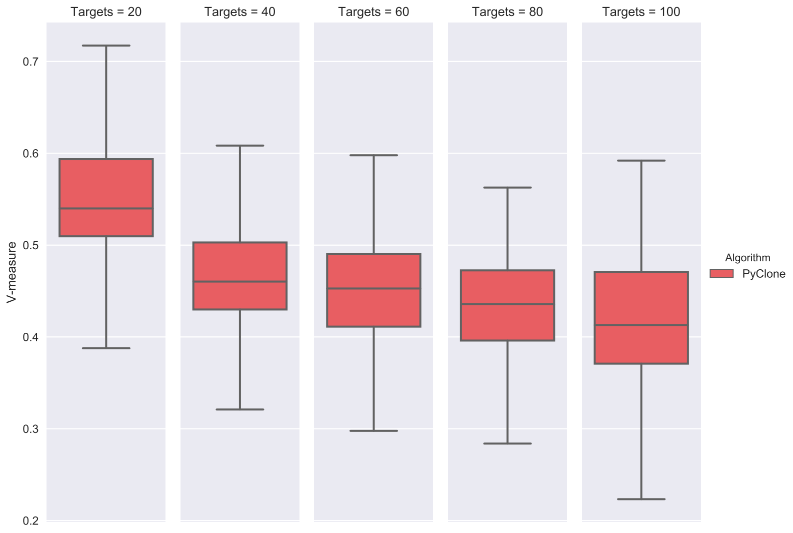

Figure 7

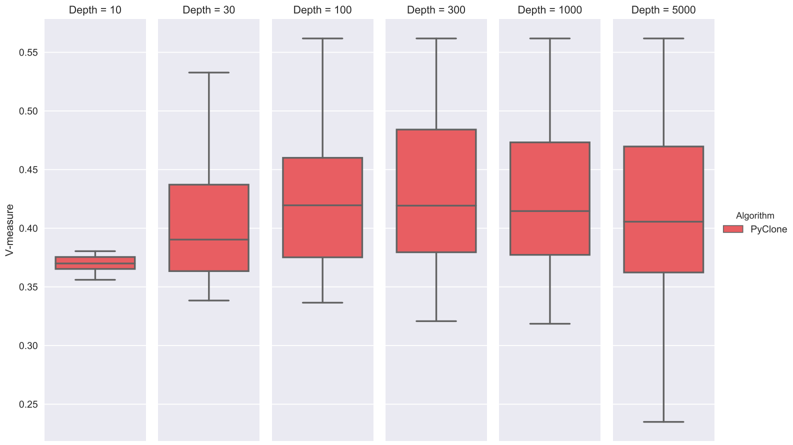

Figure 8

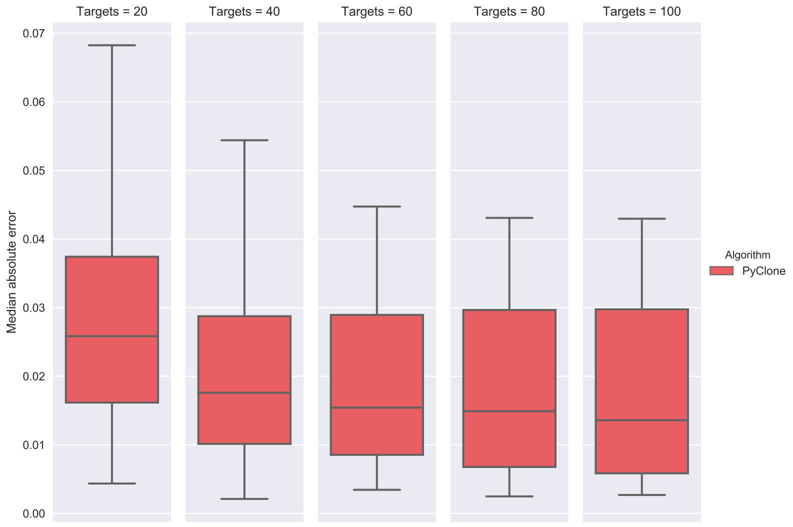

Figure 9

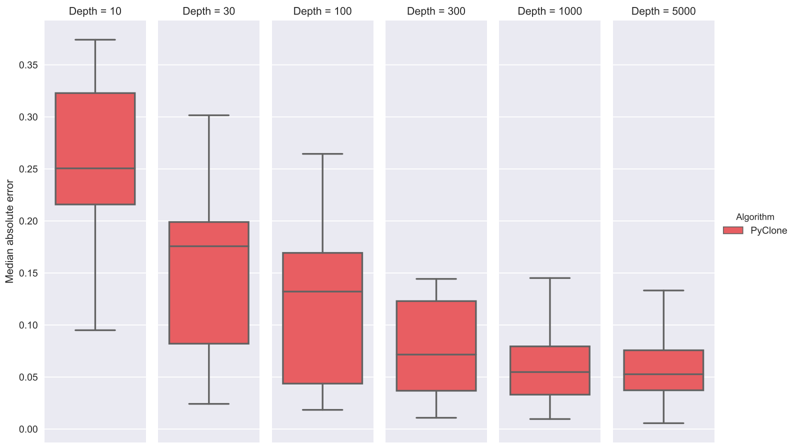

Figure 10

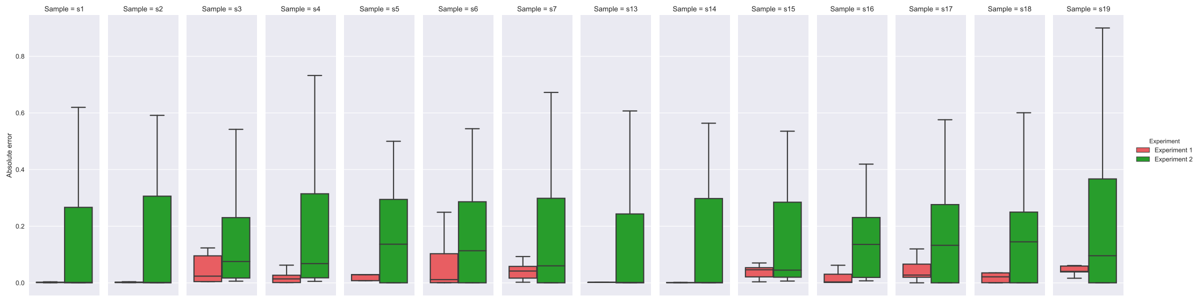

Figure 11
